# Positive Age Beliefs Lead to Lower Dementia Risk Even Among Older Persons With High-Risk Gene

**DOI:** 10.1093/geroni/igaa057.2009

**Published:** 2020-12-16

**Authors:** Becca Levy, Martin Slade, Robert Pietrzak, Luigi Ferrucci

**Affiliations:** 1 Yale School of Public Health, Woodbridge, Connecticut, United States; 2 Yale University, New Haven, Connecticut, United States; 3 Yale, New Haven, Connecticut, United States; 4 National Institute on Aging, Bethesda, Maryland, United States

## Abstract

One of the strongest risk factors for dementia is the ε4 variant of the APOE gene. Yet, many who carry it never develop dementia. The current study examined whether positive age beliefs that are acquired from the culture may reduce the risk of developing dementia among older individuals, including those who are APOE ε4 carriers. The cohort consisted of 4,765 Health and Retirement Study participants who were aged 60 or older and dementia-free at baseline. As predicted, in the total sample those with positive age beliefs at baseline were significantly less likely to develop dementia, after adjusting for relevant covariates. Among those with APOE ε4, those with positive age beliefs were 49.8% less likely to develop dementia than those with negative age beliefs. The results of this study suggest that positive age beliefs, which are modifiable and have been found to reduce stress, can act as a protective factor, even for older individuals at high risk of dementia.

